# Highlights from the Brain Conference 2026

**DOI:** 10.1093/braincomms/fcag267

**Published:** 2026-09-01

**Authors:** Manuela Marescotti

**Affiliations:** Edinburgh, UK

## Abstract

Our Scientific Editor reports the highlights of the Brain Conference 2026, which took place in London on 20 March 2026. This one-day event brought together neuroscientists from the UK and Europe to meet, hear state-of-the-art updates on neuroscientific and neurological research, and enjoy the opportunity to network.

## Graphical Abstract

**Graphical Abstract:**
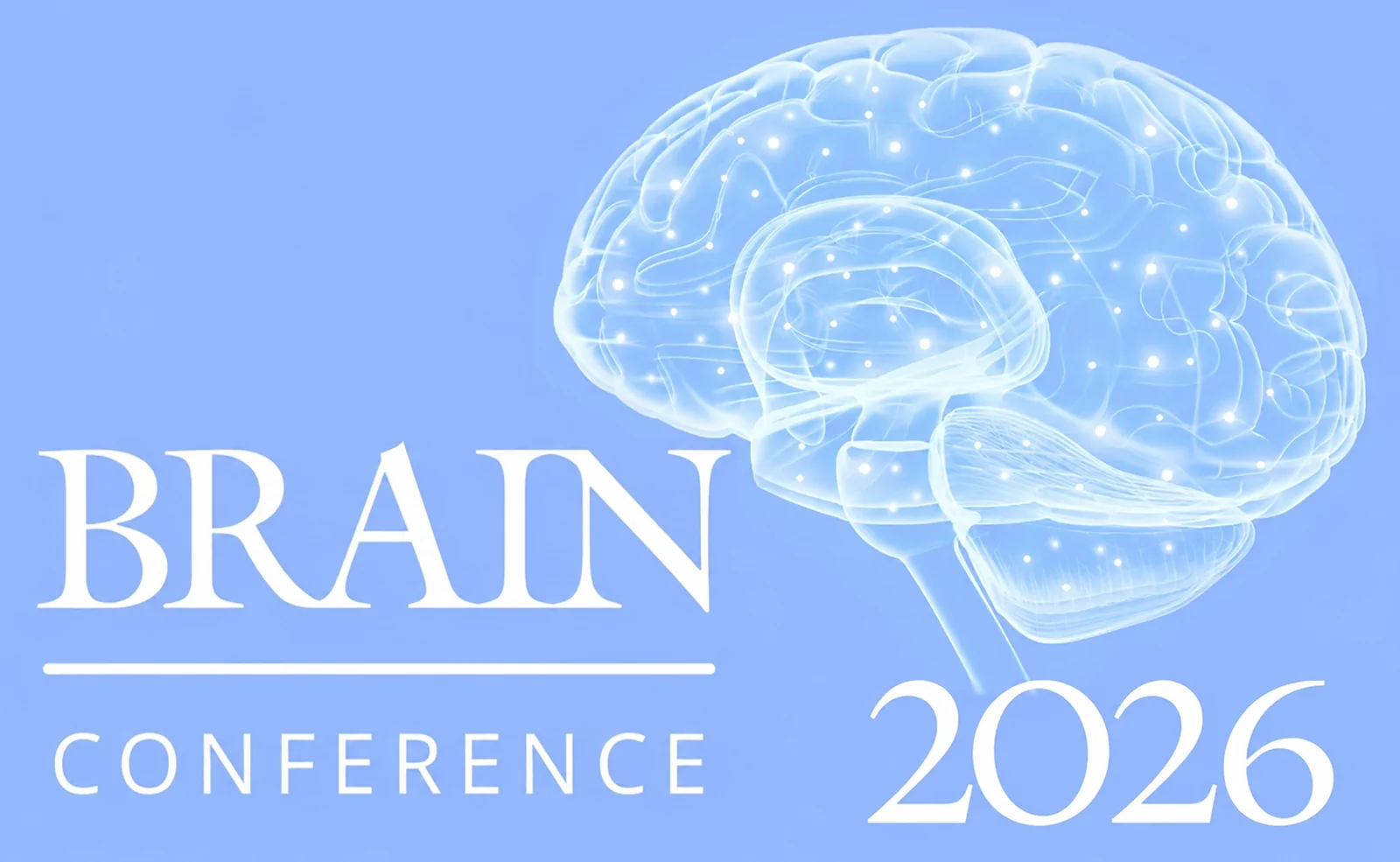

Welcome to Volume 8, Issue 5 of *Brain Communications*. On Friday, 20 March 2026, the Minster Building in London was the venue for the 6th edition of the Brain Conference (https://conference.guarantorsofbrain.org/): 17 speakers, 93 posters, 4 sessions, 250 participants, all in one day, and I was honoured to be part of the organizing committee. Since 2020^1-4^ the Brain Conference has been proving to be a highlight for the UK’s vibrant neuroscience community, bringing together high-quality neuroscience and neurology research for a day of exchanging invaluable insights and building relationships.

Throughout a dense and engaging one-day agenda, the scientific rigour and a variety of multi-disciplinary approaches shaped the sessions, which ranged from the high-profile plenary lecture to the dynamic data-blitz talks and poster presentations. The meeting was structured as follows: a plenary lecture given by the 2025 Guarantors of Brain Prize winner, three thematic sessions (‘Novel Genes and Technologies’, ‘Trauma and Regeneration’ and ‘Diseases of Later Life’), a ‘data-blitzes’ session and a poster session. The winner of the fourth *Brain Communications* Early Career Researcher (ECR) award 2025 was also announced.

The conference opened with a significant milestone: the presentation of the first Guarantors of Brain Prize. This prestigious biennial award recognizes leading national and international translational neuroscientists who have demonstrated sustained and high-level research output. The recipient is chosen through a highly competitive selection process involving both national and international panels. Professor Dimitri Kullmann (University College London) was awarded the 2025 prize. His career is a rare example of a scientist who has successfully transcended the gap between fundamental laboratory science and clinical translation. Professor Kullmann’s work has focused on the basic science of synapses and ion channels and their relationship to neurological diseases, which he has translated into pioneering genetic therapies for pharmaco-resistant epilepsy.^5^ Moreover, beyond his research, Professor Kullmann’s clinical contribution is equally distinguished; he has served for many years as a consultant manager of the medical intensive care unit at Queen Square in London, specializing in acute neurological conditions including myasthenia gravis. Lastly, Professor Reilly, chair of the Guarantors of Brain, also highlighted Professor Kullmann’s collegiate nature and commitment to mentorship, having trained multiple generations of neuroscientists.

The speakers in the ‘Novel Genes and Technologies’ session reported evidence of approaches to treat and gain insights from neuroscience conditions within the context of gene-related technologies. First, Professor William Gray (Cardiff University) led us through the promising outcomes of gene therapy delivery to the human brain via convection-enhanced delivery. Second, Dr Jonathan Cooper-Knock’s (University of Sheffield) presentation focused on the identification of genetic drivers of amyotrophic lateral sclerosis survival, specifically the gene *CCDC146*, through a machine learning-enhanced analysis of functional variants in motor neurons, and demonstrated how antisense oligonucleotide (ASO) therapy targeting this gene can rescue TDP-43 pathology and significantly extend survival by modulating cilia-mediated mechanisms. Third in this session, Professor Omer Bayraktar (Wellcome Sanger Institute) discussed the use of spatial transcriptomics to map the expression of autism risk genes during brain development, revealing that these genes are highly enriched in the thalamus and germinal zones rather than being primarily limited to the prefrontal cortex.

In the ‘Trauma and Regeneration’ session, the speakers illustrated two insightful research studies on traumatic brain injury (TBI) and one remarkable work on regenerative medicine to treat multiple sclerosis. Professor Virginia Newcombe (University of Cambridge) presented evidence on the utility of proteomic biomarkers to reduce the use of computed tomography and improve long-term prognostic accuracy in TBI. Then, Professor Adrian Liston (University of Cambridge) explained how targeted delivery of interleukin-2 can expand regulatory T-cell populations in the brain to suppress neuroinflammation effectively and reduce secondary damage across various neurological conditions, particularly in TBI. Last, Professor Denise Fitzgerald (Queen’s University Belfast) highlighted the regenerative capacity of regulatory T-cells in driving myelin repair by promoting the differentiation of oligodendrocyte progenitor cells, offering potential therapeutic avenues for multiple sclerosis.

The data blitz session started with the talk from the recipient of the *Brain Communications* ECR Award: Dr Zanna Voysey (University of Cambridge), as the first author of a remarkable research study published in *Brain Communications* in 2025, entitled ‘Sleep abnormalities are associated with greater cognitive deficits and disease activity in Huntington's disease: a 12-year polysomnographic study’.^6^ This manuscript reports a 12-year study that provided critical insights into the correlation between sleep biomarkers, such as sleep stage instability and insomnia maintenance, with the disease activity and cognitive deficits in Huntington’s disease gene carriers. I would like to take this opportunity not only to congratulate Dr Voysey for her achievement, but also to thank our scientific community and *Brain Communications* editors for their involvement and support in this multi-stage initiative.^4^ The data-blitz session speakers were selected by the Brain Conference organizers and Guarantors of Brain based on the poster abstracts submitted to the conference. These presenters, even though more junior than the invited speakers for the other sessions, and despite having a shorter time (7 min) per talk, nevertheless, contributed to the high quality of the event, presenting insights from studies covering a variety of neurological conditions and methodologies.

In the ‘Diseases of Later Life’ session, the final one for the day, the speakers reported works addressing the topic of neurodegenerative processes, not only elucidating the molecular mechanisms behind such pathological conditions but also discussing the need to optimize the protocols for trial design. In particular, Dr Robert McGeachan (University of Edinburgh) explored mechanisms underlying the accumulation of tau protein at synapses in progressive supranuclear palsy in both human patients and in animals like cats and dogs. Then, Professor Becky Carlyle (University of Oxford) reported results from a proteomic study done in her research group to identify the molecular signatures of cognitive resilience in Alzheimer’s disease. Last, Professor James Carpenter (University College London) discussed a shift toward multi-arm multi-stage (MAMS) platform trials to accelerate the evaluation of disease-modifying treatments for neurodegenerative conditions.

The enthusiasm that built up throughout the day was not lost during the final stage of the event. After the presentation of the *Brain Communications* ECR Award, Professor Masud Husain, editor-in-chief of *Brain*, introduced the new editor-in-chief of *Brain Communications*, Professor David Belin (University of Cambridge), and also awarded the best poster prize to Dr Alessandro Marrero Gagliardi (University College London) for his poster on work done on ‘U7 snRNAs based gene therapies targeting cryptic splicing events involved in ALS/FTD’. The three prizes awarded during this conference show that the core mission of the Guarantors of Brain is not only to promote quality science but also to support early-career researchers, who are often the driving force of research projects.

I am very much looking forward to the next Brain Conference in 2027.

The cover for this issue comes from He *et al.* and shows a composite of amyloid-β PET imaging and functional mapping of the default mode and dorsal attention brain networks, supporting the importance of integrating early Alzheimer's pathology with functional brain network measures to better understand the mechanisms underlying cognitive decline.^7^
